# Postnatal Serum Total Thyroxine Level Associated with Short- and Long-Term Anthropometric Outcomes in Very Preterm Infants

**DOI:** 10.3390/nu14102056

**Published:** 2022-05-14

**Authors:** Yen-Ju Chen, Wei-Ying Chu, Yu-Wen Pan, Chen-Yueh Wang, Yen-Yin Chou, Chyi-Her Lin, Kennosuke Tsuda, Osuke Iwata, Wen-Hao Yu, Yung-Chieh Lin

**Affiliations:** 1 Department of Pediatrics, National Cheng Kung University Hospital, College of Medicine, National Cheng-Kung University, Tainan 704302, Taiwan; yensweet@gmail.com (Y.-J.C.); panyuwen0527@hotmail.com (Y.-W.P.); yenyin@mail.ncku.edu.tw (Y.-Y.C.); neonate@mail.ncku.edu.tw (C.-H.L.); 2 Institute of Clinical Medicine, College of Medicine, National Cheng-Kung University, Tainan 701401, Taiwan; 3 Department of Pediatrics, Tainan Hospital, Ministry of Health and Welfare, Tainan 700007, Taiwan; raychu9629@gmail.com; 4 Department of Pediatrics, Tainan Sinlau Hospital, Tainan 701002, Taiwan; peddrwang@gmail.com; 5 Department of Pediatrics, College of Medicine, National Cheng-Kung University, Tainan 701401, Taiwan; 6 Center for Human Development and Family Science, Department of Neonatology and Pediatrics, Graduate School of Medical Sciences and Medical School, Nagoya City University, Nagoya 467-8601, Japan; kentsuda20122013@gmail.com (K.T.); o.iwata@med.nagoya-cu.ac.jp (O.I.)

**Keywords:** very preterm infants, thyroid function, anthropometric outcome, newborn screening, hypothyroxinemia, growth, developmental origins of health and disease (DOHaD)

## Abstract

Thyroxine (T4) importantly regulates the growth of newborns. Compared to fetuses with equivalent gestational ages, very preterm infants (VPIs) often experience relatively low thyroxinemia, with a normal thyroid-stimulating hormone (TSH) concentration < 10 μIU/mL. However, there is continued debate regarding postnatal thyroxine supplementation for VPIs with normal TSH and transitionally low thyroxinemia. Little research has explored the role of the postnatal total T4 (TT4) serum concentration on the growth of VPIs. In this study, we aim to clarify whether the postnatal thyroxine concentration is associated with the short- and long-term growth outcomes of VPIs. A total of 334 surviving VPIs in our previously reported cohort, born in the period August 2007–July 2016, were enrolled. The exposure variable was the postnatal TT4 concentration at 1 month old. The primary outcomes were body weight increments over 28 days after the screening and anthropometric outcomes at the corrected age of 24 months old. Infants with any hormonal replacement, severe brain injury, congenital anomaly, or cerebral palsy were excluded. In total, 290 (86.8%) VPIs were included for analysis. In the 28 days after thyroid function screening, the TT4 concentration was found to have a significant association with positive increments in body weight (mean increment: 25.7 g per 1 μg/dL; *p* < 0.001) and a positive body weight z-score (mean increment: 0.039 per 1 μg/dL; *p* = 0.037), determined by generalized estimating equation analysis. At the corrected age of 24 months old, a higher postnatal TT4 concentration was associated with a lower body mass index (mean coefficient: −0.136; 95% CI: −0.231 to −0.041, *p* = 0.005) and lower body mass index z-score (mean coefficient: −0.097; 95% CI: −0.170 to −0.024, *p* = 0.009). Infants with a TT4 concentration > 6.4 ug/dL had significantly lower odds of overweight status (odds ratio: 0.365; 95% CI: 0.177 to 0.754, *p* = 0.006). We conclude that the postnatal TT4 concentration is associated with a positive increment in body weight in the short term. At the same time, the postnatal TT4 concentration is associated with lower odds of overweight status after long-term follow-up.

## 1. Introduction

Thyroxine is a critical hormone for the growth and neurodevelopment of human newborns [1,2]. According to various national policies, newborns need to be screened and treated soon after birth for simultaneously elevated thyroid-stimulating hormone (TSH; ≥10 μIU/mL) and low thyroxine (T4) levels, which is denoted as primary congenital hypothyroidism [3,4,5]. Long-term neurodevelopmental sequela and growth retardation may be prevented if infants with primary congenital hypothyroidism are treated early and properly.

Compared with full-term infants, very preterm infants display a unique and dynamic pattern in the thyroid-related hormone profile, as prematurity is associated with incomplete development of the hypothalamic–pituitary axis [6,7]. At first, compared to fetuses at an equivalent gestational age, very preterm infants are characterized by a temporary reduction in T4 that may last for 6–8 weeks, whereas their TSH remains low to normal [7,8,9]. Second, delayed TSH elevation is a common thyroid function anomaly in very preterm infants, which is not seen in term infants [10,11,12]. Although a number of recent studies have proposed gestational age-specific references in TT4 or fT4 normal [9,13], there is still a knowledge gap regarding how to define thyroid insufficiency in very preterm infants, which is typically defined based on low T4 (<10 μg/dL) with normal TSH in term infants [14].

Hence, at present, most scholars agree that extra thyroid function screening during the hospitalization of very preterm infants is necessary [15]. After being screened, the current consensus suggests very preterm infants with elevated TSH levels (≥10.0 μIU/mL) should undergo a further follow-up, regardless of thyroxine concentration, and should be considered for treatment [11,16,17]. However, when the TSH is not elevated, the decision to initiate thyroid hormone supplementation is uncertain [7,17,18,19,20,21,22]. The effect of the temporary reduction in T4, which can last for 6–8 weeks, remains unknown in very preterm infants, who usually present rapid growth in this time window.

To date, the vast majority of works have focused on the neurodevelopmental outcomes with respect to hypothyroxinemia or the effect of hormone supplements [6,7,18,23,24,25,26] and have overlooked the effect of thyroxine on short- and long-term growth outcomes. The association between postnatal T4 and growth should be investigated before establishing a guideline for hormone supplements for very preterm infants (VPI) with low T4 and normal TSH.

In this study, we aimed to establish the relationship between the postnatal thyroxine concentration obtained at 1-month-old and short- or long-term growth outcomes by utilizing a previously reported cohort [17]. The short-term growth outcome was the bodyweight changes over 28 days after screening, while the long-term growth outcome was anthropometric parameters at the corrected age of two years.

## 2. Materials and Methods

### 2.1. Study Setting, Thyroid Function Screening Policy, and Study Design

This retrospective study aimed to identify the relationship between the total thyroxine concentration and the long-term anthropometric outcomes of VPIs born at a gestational age (GA) of 22–30 weeks. Ethical approval according to the guidelines of the Declaration of Helsinki was obtained from the Institutional Review Board of National Cheng Kung University Hospital (A-ER-106-259, 2017/11/24 and ER-98-135, 2019/6/20).

This study unit was a 20-bed tertiary neonatal intensive care unit (NICU) at the National Cheng Kung University Hospital in Tainan, Taiwan. The care volume of this unit per year is approximately 350–400 neonatal admissions, including approximately 60–70 VPIs. The clinical care policies were the same as in our previous reports [27].

**Figure 1:**
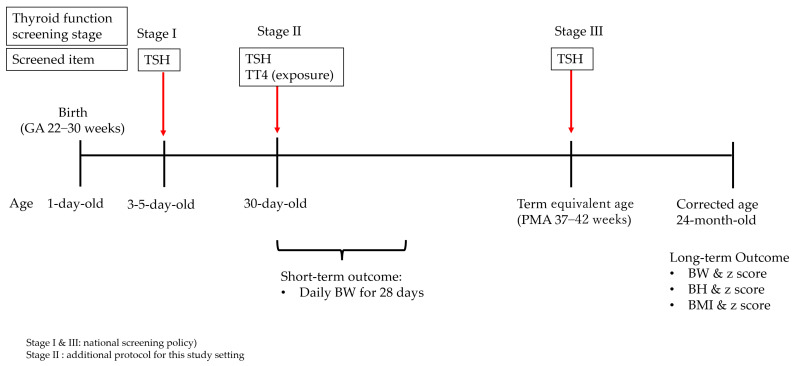
The timing scheme for thyroid function screening (red arrows) and the study design. BW: body weight; BH: body height; BMI: body mass index; GA: gestational age; PMA: postmenstrual age; TT4: total thyroxine; TSH: thyroid-stimulating hormone. Exposure indicates the exposure variable for this study. Stage I: The national screening for TSH concentration. Stage II: The local protocol for assessing the total thyroxine and TSH concentration. Stage III: The national screening is mandatory for preterm infants.

As shown in Figure 1, the study unit conducted a local screening policy (stage II) for thyroid function in very preterm infants, in addition to the national screenings at birth (stage I) and at the term-equivalent age (stage III) [10,11,17].

### 2.2. Inclusion and Exclusion Criteria

#### 2.2.1. Inclusion Criteria

VPIs born between August 2007 and July 2016 and admitted to the unit were retrospectively included by reviewing medical records in January 2019, when the participants had all completed their routine follow-up at the corrected age of 24 months. The follow-up included growth evaluation and neurodevelopment assessment. The study analysis was conducted in April 2019.

#### 2.2.2. Exclusion Criteria

- Maternal thyroid disease during pregnancy;
- Age > 7 days old at admission;
- Death at discharge;
- Severe brain injury, such as severe intraventricular hemorrhage (IVH) or periventricular leukomalacia (PVL), which may affect the hypothalamic–pituitary axis and thyroid function [6];
- Congenital anomalies or syndromic gene anomalies;
- Patients with any event of elevated TSH (≥10 μIU/mL) during serum or blood spot tests with/without thyroid hormone therapy at any stage;
- Treated with Levothyroxine;
- Total thyroxine and TSH data are not simultaneously available at stage II screening;
- Cerebral palsy, which may affect the measurement of anthropometry.

### 2.3. Independent Variable: Thyroid Function Data

The total thyroxine concentration in the serum at the postnatal age of one month was defined as the exposure. The total thyroxine obtained at stage II (Figure 1) by radioimmunoassay was retrieved from medical records [17].

### 2.4. Clinical Variables and Covariates

Maternal education level, gestational-related diseases, and medication history were reviewed and recorded in birth charts. The perinatal data included GA, body weight at birth, sex, the z-score of the birth body weight, Apgar scores at 1 and 5 min after birth, the method of delivery, and delivery room interventions. Data on respiratory distress syndrome (RDS), patent ductus arteriosus (PDA), sepsis, intraventricular hemorrhage (IVH), necrotizing enterocolitis (NEC), retinopathy of prematurity (ROP), postnatal steroids therapy, chronic lung disease (CLD) [28], periventricular leukomalacia (PVL), postnatal steroid therapy for CLD, postnatal age, and postmenstrual age were obtained by reviewing the discharge notes.

Postnatal age and postmenstrual age at the screening, the serum TSH concentration, and the results of the two national TSH screenings were recorded. The Fenton growth chart was used to calculate the anthropometric z-score for the hospitalized stay [29], and the post-discharge anthropometric z-score was calculated according to the standard established and released by the World Health Organization (WHO) [30]. Weight statuses were defined by the Centers for Disease Control and Prevention, USA, as follows: underweight status (zBMI < −1.645 or < 5th percentile), overweight status (zBMI ≥ 1.036 or ≥ 85th percentile), and obese status (zBMI ≥ 1.645 or ≥ 95th percentile) [31].

### 2.5. Outcomes

#### 2.5.1. Primary Outcome

- Short-term growth: The increment in body weight and the z-score of body weight over 28 days after the screening in stage II were defined as the short-term outcomes.
- Long-term growth: The BMI z-score and weight status at the corrected age of 24 months were defined as the long-term outcomes.

#### 2.5.2. Secondary Outcomes

Other anthropometric variables at the corrected age of 24 months were considered secondary outcomes, including body weight (BW), body height (BH), body mass index (BMI), the z-score of body weight (zBW), and the z-score of body height (zBH).

### 2.6. Statistical Analysis

All the analyses were conducted in SPSS (version 26, IBM, Armonk, NY, USA). The dependence of the anthropometric variables on the clinical variables and total thyroxine was first assessed by univariate analysis after adjusting for variables chosen *a priori*. Multivariable linear regression was performed to assess the additive effect of individual anthropometric measurements (including body weight and z-score), adjusting for potential confounding variables. A generalized estimating equation (GEE) was used for repeated measures analysis of the longitudinal anthropometric data. In the GEE model, a clinical risk having significant coefficients with an anthropometric variable repeated during follow-up was interpreted as a factor associated with growth increments.

The thyroxine level was divided into a four-level categorical variable indicating the quartiles. Adjusted relationships were evaluated using a logistic regression model in order to evaluate the associations between the increment in body weight and the thyroxine level. The results were expressed as adjusted odds ratios (aOR) and had a 95% confidence interval (CI). Graphs were illustrated using GraphPad Prism (version 9.0.0 for Windows, GraphPad Software, San Diego, CA, USA). The results were considered statistically significant if a *p*-value was less than 0.05.

## 3. Results

### 3.1. The Enrollment of Neurologically Intact Survivors with Complete Thyroid Function Profiles during Hospitalization

**Figure 2:**
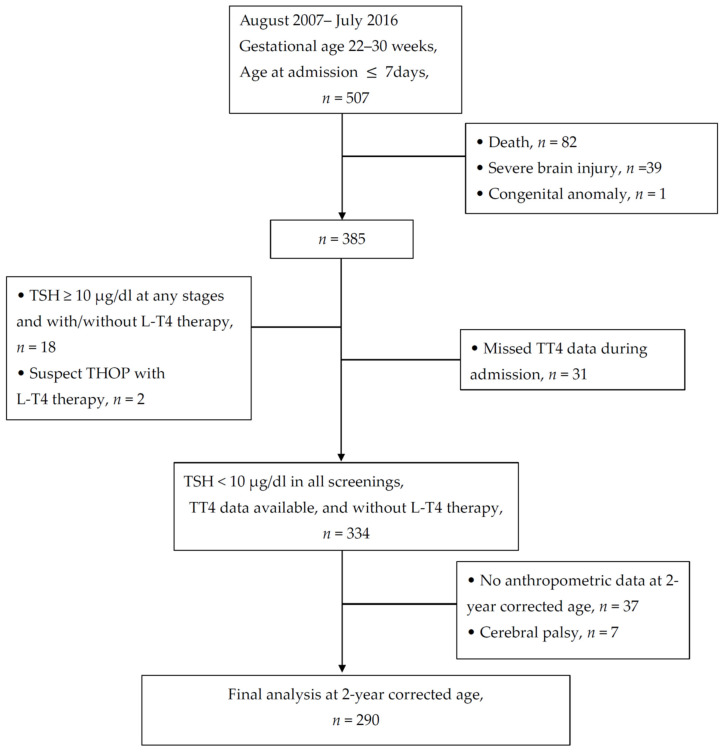
Flow chart of patient enrollment. L-T4: levothyroxine; THOP: transient hypothyroidism of prematurity; TT4: total serum thyroxine; TSH: thyroid stimulation hormone.

A total of 290 VPIs were finally analyzed to test the hypothesis of this study (Figure 2).

The mean gestational age was 27.6 weeks, and the mean birth body weight was 1038 g. The mean age of the stage II thyroxine screening was 29.9 days. The thyroxine concentration (µg/dL) was 6.58 ± 1.95 (mean ± standard deviation), with a range of 1.30–14.02, a median of 6.40, and an interquartile range of 5.28–7.85 (Table 1).

### 3.2. The Association between Serum Total Thyroxine Concentration and the 28-Day Growth Increment after Stage II Screening

Through daily observation after the stage II screening of body weight and body weight z-score over 28 days, a higher total thyroxine concentration was associated with a higher body weight increment and higher body weight z-score increment (Table S1 and Table 2).

**Figure 3:**
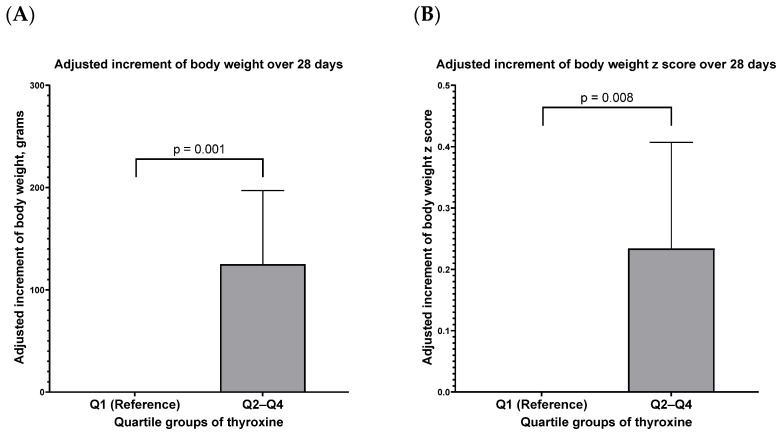
The quartiles of thyroxine concentration and their association with the 28-day increments in body weight (**A**) and body weight z-score (**B**). Adjusted for gestational age, sex, prenatal steroid, small for gestational age, and respiratory distress syndrome requiring surfactant use. Thyroxine group: Q1: ≤ 5.28; Q2: 5.29–6.40; Q3: 6.41–7.85; Q4: > 7.85 μg/dL (detailed statistics in Supplementary Table S3).

Using infants within the lowest quartile (Q1: thyroxine level ≤ 5.28 μg/dL) in this study as a reference, infants with total thyroxine in Q2–Q4, compared to infants with total thyroxine in Q1, had a significantly higher body weight increment and higher body weight z-score increment over the 28 days of observation after the screening at stage II (Figure 3, Table S3).

### 3.3. Dependence of the 24-Month Z-Score of BMI on Clinical Variables and Thyroxine

The 290 infants returned to follow-up at the clinic at 24.1 ± 0.9 months of their corrected age. The anthropometric variables at 24 months of corrected age are shown in Table S4. Exploratory models initially showed the non-significant dependence of the 24-month z-score of BMI on thyroxine concentration; however, significance was observed after gradually increasing the covariates of the models (models I–III, Table S5). In the additional adjusted models (models 1–3, Table 3), which had more covariates associated with zBMI, the results still showed a significantly negative correlation between thyroxine concentration and zBMI (*p* = 0.006 and 0.009, respectively, for models 2 and 3). However, unlike BMI and zBMI, the total thyroxine concentration in postnatal serum had no association with BW, BH, zBW, or zBH at the corrected age of 24 months (Tables S6 and S7).

### 3.4. Lower Postnatal Thyroxine Concentrations Associated with Overweight Status at the Corrected Age of 24 Months

As postnatal thyroxine had a negative association with body weight’s z-score at the CA of 24 months, we studied the relationship between the thyroxine quartile and weight status (Table 4). Of the 290 infants, 22 (7.6%) infants were underweight, 46 (15.9%) infants were overweight, and 16 (5.5%) were obese at the corrected age of 24 months.

We examined the relationship between weight status and postnatal thyroxine concentration. We observed that infants with a higher thyroxine quartile had lower odds of being overweight. After adjustment with a clinical variable that was preliminarily chosen *a priori,* infants with a thyroxine concentration within Q3–Q4 had significantly lower odds of being overweight than infants with a thyroxine concentration within Q1–Q2 (mean aOR: 0.358; *p* = 0.005); see Table 4.

By adjusting with more clinical variables in the multivariate regression model, we further confirmed that infants with thyroxine concentration within Q3–Q4 had a significantly lower odds ratio of reaching overweight status than infants with a thyroxine concentration within Q1–Q2 (aOR = 0.365; *p* = 0.006); see Table 5. A higher postnatal thyroxine concentration was significantly associated with lower odds of reaching overweight status (aOR = 0.782; *p* = 0.022).

## 4. Discussion

In this study, we used a previously reported cohort of very preterm infants to determine the respective associations between the postnatal total thyroxine level and short- and long-term growth. Our study showed that the postnatal total thyroxine concentration obtained at one-month-old in very preterm infants had a significantly positive correlation with the body weight increment over the 28 days following the stage II screening. Furthermore, a higher postnatal total thyroxine concentration was linked to a lower BMI z-score and lower odds of reaching overweight status at the corrected age of two years. Taken together, these findings suggest that postnatal total thyroxine levels are essential for the growth of very preterm infants. However, to define the optimal total thyroxine level for growth, more research is required.

### 4.1. Extra-Uterine Growth of Very Preterm Infants and Postnatal Thyroxine Concentration

At present, an optimal growth velocity of 14–20 g/kg/day is recommended when very preterm infants receive adequate nutrition [32,33,34]. In the very preterm infants in our study, the mean growth velocity after the stage II thyroid function screening was 21.5 g/day, which was within the suggested range. According to our estimates, our studied infants gained a mean weight of about 600 g within the 28-day observation period. In our data, each increment in the unit (ug/dL) of thyroxine contributed to a mean 25.7-g increment within the 28-day period of observation, which is estimated to be 4.2% of 600 g. Compared to infants with thyroxine levels in Q1, infants with thyroxine levels in Q2 had a higher average body increment of 165.7 g, estimated to be 27.5% of 600 g over a 28-day period (Table S2). Hence, we regard that the thyroxine level may be an essential factor for growth in very preterm infants during hospitalization.

Human fetuses rely on maternal thyroid hormones from early gestation. This reliance then decreases and remains at low levels until birth [35,36]. Cord blood thyroid hormones are correlated with maternal physical condition and thyroid hormone levels [37,38,39]. However, we could not compare our novel finding to published references, as few attempts were made to investigate the role of single postnatal thyroxine levels in contributing to the growth of hospitalized, very preterm infants. The positive correlations between thyroxine and growth increments in our study could be inferred from observational studies about the maternal–fetus interaction. In population research, maternal hypothyroxinemia is also correlated with fetus intra-uterine growth retardation [40,41,42,43,44,45,46]; furthermore, thyroid hormone therapy among pregnant women with sub-clinical hypothyroidism is associated with a decreased risk of low birth weight in their offspring [47].

We also found infants in Q2, Q3, or Q4 had a similar short-term growth increment (Table S2), which implies that higher thyroxine is not necessarily related to better short-term growth. From a thyroxine supplementation trial in extremely preterm infants [22], thyroxine supplementation, regardless of the baseline level, contributed to higher free T4 concentration but not to better growth parameters at a term-equivalent age. Based on our findings, we assumed that the short-term growth of infants might improve once the level is higher than a kind of physiologic level.

Due to the limited subject number, we could not determine and provide an optimal cut-point for the initiation of thyroxine supplementation at 1 month in very preterm infants to benefit their short-term growth. However, our study provides an important clue for monitoring thyroid function in very preterm infants with growth faltering.

### 4.2. Long-Term Anthropometry and Thyroxine Concentration in Early Life

In this study, we observed that the single postnatal thyroxine concentration in early life was an inversely independent factor for long-term anthropometric variables, including zBMI, BMI, and BW, after adjusting for neonatal morbidities associated with prematurity. However, there is limited research investigating the relationship between postnatal thyroxine concentration and long-term growth in children born very preterm. To the best of our knowledge, our study was the first to report that a lower risk of overweight status at 24 months old is associated with a higher thyroxine concentration at the neonatal stage. Although our study only used a single concentration, this time point may adequately represent the thyroxine level in early life [9].

An observation of the published anthropometric reports considering term infants who received appropriate treatment for congenital hypothyroidism may help with interpreting our results. Term infants receiving appropriate treatment for congenital hypothyroidism had different levels of growth impairment at 24 months old, with their body height being more impaired than body weight (23% vs. 13–15%) [48]. Compared to the general population, the rate of BMI, overweight status, or obesity at 24 months of age was reported being high in children with properly treated CH [49,50,51]. Combining the results from term infant studies and our findings, we inferred that the thyroid hormone might exert its effects on growth during the fetal period.

Preterm birth results in interrupted thyroid hormone supply from the mother [35,36], which leads to preterm infants having relatively higher rates of hypothyroxinemia compared to fetuses at an equivalent gestation age. Our study implied that the relatively higher rate of hypothyroxinemia during the first month of life might be associated with long-term growth in very preterm infants. Future research should address these important findings in more detail, particularly with regard to the role of thyroxine in the developmental origins of health and disease (DOHaD).

### 4.3. The Optimal Total Thyroxine Concentration for Very Preterm Infants

Thyroxine is an essential hormone regulating growth and metabolic rate. Mother–infant dyad studies, which demonstrate both maternal hypothyroidism and hyperthyroidism resulting in small for gestational age infants, indicate the importance of an optimal balance of the thyroid hormone [44,45,47,52]; however, the optimal value for preterm infants has not yet been elucidated [13], and hormone intervention in preterm infants may be interfered with by the therapeutic guidelines for infants with congenital hypothyroidism. For infants under levothyroxine therapy, the therapeutic target concentration of total thyroxine for congenital hypothyroidism is 10–16 µg/dL [53]. The value of total thyroxine in this study cohort at 1-month-old was 6.6 ± 2.0 µg/dL, which is much less than this recommended level. Thus, applying the therapeutic target concentration for congenital hypothyroidism to low thyroxinemia in very preterm infants seems unsuitable.

Hence, our results may provide a strong concept for the management of thyroxine levels of very preterm infants, especially those with low total T4 levels and growth faltering (Tables S1–S3). From Figure 3, we can observe the comparison in growth increment among infants with total thyroxine within Q2–Q4 and Q1. We hypothesize that infants may reach an adequate short-term growth rate once their total thyroxine reaches a physiologic threshold (Table S3). However, the level (Q1) may be limited to this study and may not be generalized to other study populations with various enrollment criteria. In summary, our study preliminarily provided important evidence for clinicians to conduct more future research in order to determine the level at which to initiate and maintain hormone supplementation for the growth of very preterm infants.

### 4.4. Strengths and Limitations

The strengths of this study were the relatively large number of VPIs (*n* = 331) in the cohort and a high follow-up rate (90.3%) at 24 months. In addition, we considered the multiple factors of the comorbidities associated with prematurity and how they relate to long-term growth. We acknowledge the absence of nutritional intake information during hospitalization as a limitation. However, all VPIs in this study received a similar nutrition protocol during this period as in our previous report [54].

Our findings may not be generalizable beyond the study criteria. Our data set was limited to VPIs whose serial TSH concentrations were normal (<10 µg/dL), without thyroid hormone therapy and without brain injury. A single point of TT4 may be another weak point, and not using free T4 may be another one. The authors agreed with the important concept of using serial thyroid function screening to decipher the optimal trajectory of thyroxine concentration and its association with growth outcomes, as proposed by William et al. [55].

As we only examined total T4 concentration, not free T4 concentration, and did not include blood binding proteins (e.g., thyroxine-binding protein, prealbumin, albumin), one should be cautious when interpreting the study results. However, many studies showed a significant correlation between total T4 and free T4 values; in particular, free T4 correlates highly with total T4 in primary hypothyroidism. Hence, laboratory standards will be important in considering which low birth weight infants with “true” hypothyroxinemia are included among the patients with apparent hypothyroxinemia [56,57].

## 5. Conclusions

In very preterm infants, the thyroxine concentration at the age of 1 month has a significant correlation with the short-term increment in body weight following screening. As for the long-term anthropometric outcomes, infants with higher postnatal thyroxine levels had lower odds of reaching overweight status at the corrected age of 24 months. Considering that thyroxine is a critical hormone in the important growth window of very preterm infants, future research will further investigate the role of postnatal thyroxine on the growth of very preterm infants.

## Figures and Tables

**Table 1:** Background clinical characteristics of the study cohort.

*N* for Analysis	290
**Maternal and antenatal variables**	
Maternal age, years	32.1 ± 4.8
Maternal education level (≥ college), *n* (%)	170 (58.6)
Antenatal steroid, *n* (%)	255 (87.9)
Pre-eclampsia, *n* (%)	58 (20)
Gestational diabetes mellitus, *n* (%)	12 (4.1)
**Variables in perinatal period**	
Gestational age, weeks	27.6 ± 1.9
Body weight at birth, grams	1030 ± 242
Z-score of body weight at birth	−0.210 ± 0.749
Sex, male, *n* (%)	159 (54.8)
Method of delivery, Cesarean section, *n* (%)	166 (57.2)
Inborn, *n* (%)	248 (85.5)
Multi-gestation, *n* (%)	81 (27.9)
Resuscitation at birth (Intubation), *n* (%)	21 (7.2)
Apgar score at 5 min, median (IQR)	8 (7–9)
Early onset sepsis, *n* (%)	7 (2.4)
**Variables during hospital stay**	
Surfactant-treated respiratory distress syndrome, *n* (%)	75 (25.9)
Late-onset sepsis, *n* (%)	49 (16.9)
Necrotizing enterocolitis (stage ≥ 2), *n* (%)	13 (4.5)
Treated retinopathy of prematurity, *n* (%)	23 (7.9)
Chronic lung disease (CLD), *n* (%)	89 (30.7)
Postnatal steroid for CLD, *n* (%)	25 (8.6)
**Variables at thyroid function screening at stage II**	
Postnatal age, days	29.9 ± 4.9
Postmenstrual age, weeks	31.9 ± 1.9
Serum thyroid-stimulating hormone, μIU/mL	3.42 ± 1.94
Serum total thyroxine concentration, μg/dL;	6.58 ± 1.95
**Variables at discharge**	
Postnatal age, days	67.2 ± 25.6
Postmenstrual age at discharge, weeks	37.1 ± 2.5

Variables are described as numbers (percentage), mean ± standard deviation, and median (lower–upper quartiles).

**Table 2:** The association between the thyroxine concentration at 1 month old and the outcomes for the following 28-day increments of body weight and body weight z-score (multivariate analysis).

		Increment in Bodyweight, Gram			Increment of Bodyweight z-Score		
	Reference	Mean	95% CI	*p*-Value	Mean	95% CI	*p*-Value
Time after the screening, day		21.5	20.3, 22.8	**<0.001**	−0.018	−0.02, −0.015	**<0.001**
GA, week		145.8	129.1, 162.5	**<0.001**	−0.093	−0.133, −0.053	**<0.001**
SGA	no	−419.3	−503.5, −335.1	**<0.001**	−1.261	−1.484, −1.039	**<0.001**
Sex	female	150.3	93.8, 206.8	**<0.001**	0.074	−0.066, 0.215	0.300
Prenatal steroid	no	36.1	−39.9, 112.0	0.352	0.090	−0.110, 0.290	0.379
Surfactant for RDS	no	−23.7	−90.3, 42.9	0.486	−0.073	−0.247, 0.102	0.414
TT4, μg/dL		25.7	11.3, 40.2	**<0.001**	0.039	0.002, 0.075	**0.037**

CI: confidence interval; GA: gestational age; SGA: small for gestation age; RDS: respiratory distress syndrome; TT4: total serum thyroxine. Multivariate analysis by generalized estimating equations was performed for each dependent variable and independent variable. Statistical significance was assumed for *p* < 0.05 (indicated in bold).

**Table 3:** Primary outcome: the dependence of body mass index z-scores at 24 months old on clinical variables (multivariate analyses).

	Multivariate Model 1			Multivariate Model 2			Multivariate Model 3		
	β	95% CI	*p*	β	95% CI	*p*	β	95% CI	*p*
Gestational age	0.063	−0.031, 0.156	0.187				0.116	0.021, 0.210	**0.016**
Birth body weight				0.001	0.001, 0.002	0.001			
z-score of birth body weight							0.448	0.247, 0.650	**<0.001**
Small for gestational age	−0.470	−0.958, 0.019	0.060	−0.127	−0.631, 0.377	0.619			
Sex	−0.044	−0.312, 0.224	0.744	−0.145	−0.415, 0.126	0.293	−0.042	−0.303, 0.219	0.752
Pre-eclampsia	−0.400	−0.756, −0.044	0.028	−0.264	−0.611, 0.083	0.135	−0.157	−0.523, 0.21	0.401
Surfactant for RDS	−0.152	−0.466, 0.162	0.341	−0.143	−0.445, 0.158	0.351	−0.187	−0.492, 0.118	0.228
Surgery for PDA	−0.200	−0.605, 0.204	0.331	−0.111	−0.506, 0.284	0.581	−0.174	−0.567, 0.219	0.385
Surgery for ROP	−0.346	−0.886, 0.195	0.209	−0.238	−0.769, 0.292	0.377	−0.252	−0.78, 0.276	0.348
Postnatal steroid	−0.601	−1.128, −0.074	**0.026**	−0.488	−0.998, 0.022	0.060	−0.489	−1.001, 0.024	0.061
Total thyroxine, μg/dL	−0.074	−0.148, 0.000	**0.050**	**−0.104**	**−0.177, −0.03**	**0.006**	−0.097	−0.170, −0.024	**0.009**

Linear regression was performed for each dependent variable and independent variable. RDS: respiratory distress syndrome; PDA: hemodynamic significant patent ductus arteriosus; ROP: retinopathy of prematurity; CI: confidence interval. Multivariate logistic regression was performed for each dependent variable and independent variable. Model 1 adjusted for the gestational age and z-score of the birth body weight. Model 2 adjusted for birth body weight and the z-score of the birth body weight. Model 3 adjusted for the variables of body weight and small for gestational age. Statistical significance was assumed for *p* < 0.05 (indicated in bold).

**Table 4:** Dependence of weight status at 24 months corrected age on different thyroxine concentration quartiles.

Weight Status Category		Underweight (BMI < 5 Percentile)			Overweight (BMI ≥ 85 Percentile)			Obese (BMI ≥ 95 Percentile)		
Thyroxine Concentration Quartiles	Ref.	aOR	95% CI	*p*	aOR	95% CI	*p*	aOR	95% CI	*p*
Q2–Q4	Q1	0.457	0.160, 1.304	0.143	0.342	0.040, 2.894	0.342	0.387	0.111, 1.351	0.387
Q3–Q4	Q1–Q2	1.065	0.378, 3.304	0.146	0.358	0.174, 0.737	**0.005**	0.498	0.165, 1.506	0.498
Q4	Q1–Q3	1.078	0.320, 3.635	0.136	0.839	0.387, 1.781	0.633	0.797	0.236, 2.689	0.715

Ref: reference, BMI: body mass index, aOR: adjusted odds ratio with gestational age, sex, birth body weight’s z-score, and postnatal steroid therapy. Q1: ≤5.28; Q2: 5.29–6.40; Q3: 6.41–7.85; Q4: >7.85 μg/dL. Multivariate logistic regression was performed for each dependent variable and independent variable. Statistical significance was assumed for *p* < 0.05 (indicated in bold).

**Table 5:** Association between thyroxine level and overweight status at the corrected age of 24 months.

		Multivariate Model 1			Multivariate Model 2		
	Reference	aOR	95% CI	*p*	aOR	95% CI	*p*
Gestational age		1.340	1.019, 1.761	**0.036**	1.343	1.026, 1.757	**0.032**
Z-score of birth body weight		2.535	1.385, 4.64	**0.003**	2.574	1.401, 4.728	**0.002**
Sex	female	0.821	0.416, 1.62	0.570	0.832	0.422, 1.639	0.594
Pre-eclampsia	No	0.687	0.224, 2.105	0.511	0.716	0.234, 2.19	0.558
Surfactant for RDS	No	0.993	0.433, 2.274	0.986	0.989	0.432, 2.264	0.979
Surgery for PDA	No	1.059	0.338, 3.315	0.922	1.084	0.345, 3.408	0.890
Surgery for ROP	No	0.353	0.039, 3.172	0.352	0.418	0.047, 3.695	0.433
Postnatal steroid	no	0.397	0.046, 3.456	0.402	0.382	0.043, 3.356	0.385
Total thyroxine, μg/dL		0.782	0.633, 0.966	**0.022**			
Total thyroxine, Q3–Q4	Q1–Q2				0.365	0.177, 0.754	**0.006**

Overweight defined by the CDC (BMI z-score ≥ 85th percentile). aOR: adjusted odds ratio; RDS: respiratory distress syndrome; PDA: patent ductus arteriosus; ROP: retinopathy of prematurity. Q1: ≤5.28; Q2: 5.29–6.40; Q3: 6.41–7.85; Q4: >7.85 μg/dL. Multivariate logistic regression was performed for each dependent variable and independent variable. Statistical significance was assumed for *p* < 0.05 (indicated in bold).

## Data Availability

The corresponding author had full access to the data set used and analyzed during the current study. The data sets used during the current study are available from the corresponding author on reasonable request.

## Supplementary Materials

The following supporting information can be downloaded at: https://www.mdpi.com/article/10.3390/nu14102056/s1, Table S1: Variates associated with body weight increment and body weight z-score increment over the 28 days after the 1-month thyroid function screening (Univariate analyses); Table S2: Variates associated with body weight increment and body weight z-score increment over the 28 days after the 1-month thyroid function screening (Multivariate analyses); Table S3: Variates associated with body weight increment and body weight z-score increment over the 28 days after the 1-month thyroid function screening (Table S2 Continued); Table S4: The anthropometric variables by genders at 24 months corrected age; Table S5: Primary outcome: dependence of body mass index z-score at 24 months on clinical variables—univariate analysis and multivariate analyses; Table S6: Secondary outcome: dependence of body mass index, body weight, and body height at 24 months on clinical variables—multivariate analyses; Table S7: Secondary outcome: dependence of body weight z-scores and body height z-score at 24 months on clinical variables—multivariate analyses.
