# Supplementary material for: Postnatal Serum Total Thyroxine Level Associated with Short- and Long-Term Anthropometric Outcomes in Very Preterm Infants
Source: Nutrients. 2022 May 14;14(10):2056. doi: 10.3390/nu14102056 (PMC9143878; doi:10.3390/nu14102056)
Supplement: Supplementary file 1 [file nutrients-14-02056-s001.zip › nutrients-1677113-supplementary.pdf]

Supplemental table S1. Variates associated with body weight increment and body weight z-score increment over the 28 days after the 1-month thyroid function screening. (Univariate analyses)

		Body weight increment, gram			Body weight z score increment		
	reference	mean	95%CI	*p*	mean	95%CI	*p*
Time after the screening, day		18.6	17.0, 20.2	**<0.001**	-0.017	-0.02, -0.014	**<0.001**
GA, week		150.5	133.6, 167.5	**<0.001**	-0.096	-0.141, -0.052	**<0.001**
SGA	no	-328.7	-465.6, -191.7	**<0.001**	-1.356	-1.602, -1.11	**<0.001**
Sex	female	149.3	56.4, 242.1	**<0.001**	0.056	-0.117, 0.229	0.526
Prenatal steroid	no	-60.7	-195.3, 73.8	0.376	0.102	-0.145, 0.349	0.418
Surfactant for RDS	no	-203.6	- 308.7, -98.6	**<0.001**	-0.052	-0.246, 0.143	0.604
TT4, μg/dL		79.0	57.1, 100.8	**<0.001**	0.009	-0.036, 0.054	0.707
TT4, Quartile		131.4	92.3, 170.5	**<0.001**	0.009	-0.069, 0.087	0.816

GA: gestational age; SGA: small for gestational age; RDS: respiratory distress syndrome; TT4: total thyroxine serum level. Univariate analysis by generalized estimating equations was performed for each dependent variable and inde-pendent variable. Statistical significance was assumed for $p < 0.05$ (indicated in bold).

Supplemental table S2. Variates associated with body weight increment and body weight z-score increment over the 28 days after the 1-month thyroid function screening. (Multivariate analyses)

		Body weight increment, gram			Body weight z score increment		
	reference	mean	95%CI	*p*	mean	95%CI	*p*
Time after the screening, day			20.4, 22.8	**<0.001**	-0.017	-0.02, -0.015	**<0.001**
GA		145.2	128.8, 161.7	**<0.001**	-0.096	-0.135, -0.056	**<0.001**
SGA		-420.0	-510.3, -329.7	**<0.001**	-1.256	-1.484, -1.028	**<0.001**
Sex	female	163.2	106.4, 219.9	**<0.001**	0.107	-0.033, 0.247	0.134
Prenatal steroid	no	31.3	-41.9, 104.6	0.401	0.068	-0.13, 0.267	0.501
Surfactant for RDS	no	-28.4	-94.6, 37.9	0.377	-0.082	-0.254, -0.089	0.346
TT4, Quartile							
TT4, Q2	Q1	165.7	77.6, 253.8	**<0.001**	0.283	0.069, 0.496	**0.009**
TT4, Q3	Q1	78.9	-8.7, 166.5	0.077	0.146	-0.062, 0.355	0.168
TT4, Q4	Q1	126.7	49.8, 203.7	**0.001**	0.314	0.121, 0.508	**0.001**

GA: gestational age; SGA: small for gestational age; RDS: respiratory distress syndrome; TT4: total thyroxine serum level. Multivariate analysis by generalized estimating equations was performed for each dependent variable and inde-pendent variable. Statistical significance was assumed for $p < 0.05$ (indicated in bold). Q1: <= 5.28 ; Q2: 5.29 - 6.40 ; Q3: 6.41 - 7.85 ; Q4: >7.85 µg/dL

Supplemental table S3. Variates associated with body weight increment and body weight z-score increment over the 28 days after the 1-month thyroid function screening. (Table S2 Continued)

		Body weight increment, gram			Body weight z score increment		
	reference	mean	95%CI	*p*	mean	95%CI	*p*
Time after the screening, day		21.5	20.3, 22.8	**<0.001**	-0.018	-0.02, -0.015	**<0.001**
GA		146.3	130.3, 162.2	**<0.001**	-0.097	-0.135, -0.058	**<0.001**
SGA		-421.7	-506.8, -336.6	**<0.001**	-1.259	-1.484, -1.033	**<0.001**
Sex	female	156.2	98.9, 213.5	**<0.001**	0.092	-0.049, 0.234	0.202
Prenatal steroid	no	23.2	-51.3, 97.7	0.541	0.068	-0.131, 0.267	0.503
Surfactant for RDS	no	-22.9	-88.4, 42.5	0.492	-0.074	-0.246, 0.097	0.396
TT4, Quartile							
Q2–Q4	Q1	125.6	54.0, 197.2	**0.001**	0.234	0.062, 0.407	**0.008**

GA: gestational age; SGA: small for gestational age; RDS: respiratory distress syndrome; TT4: total thyroxine serum level. Multivariate analysis by generalized estimating equations was performed for each dependent variable and inde-pendent variable. Statistical significance was assumed for $p < 0.05$ (indicated in bold). Q1: <= 5.28 ; Q2: 5.29 - 6.40 ; Q3: 6.41 - 7.85 ; Q4: >7.85 µg/dL

Supplemental table S4. The anthropometric variables by genders at 24 months corrected age.

	Boy	Girl
Numbers	159	131
Weight, kg	11.7 ± 1.7	11.1 ± 1.5
Height, cm	86.1± 3.6	84.9 ± 3.4
BMI, kg/m2	15.8 ± 1.6	15.4 ± 1.5
Body weight z score	-0.44 ± 1.26	-0.42 ±1.14
Body height z score	-0.58 ± 1.17	-0.51 ± 1.06
BMI z score	-0.13 ± 1.22	-0.17 ± 1.10

BMI: body mass index; data are described as mean ± standard deviation.

Supplemental Table S5. Primary outcome: dependence of body mass index z-scores at 24 months old on clinical variables: univariate analysis and multivariate analyses

	Univariate			Multivariate Model I			Multivariate Model II			Multivariate Model III		
	β	95%CI (LB, UB)	*p*	*β*	95%CI (LB, UB)	*p*	*β*	95%CI (LB, UB)	*p*	*β*	95%CI (LB, UB)	*p*
Gestational age	0.089	(0.018, 0.160)	**0.014**									
Birth body weight	0.143	(0.090, 0.197)	**<0.001**	0.187	(0.128, 0.247)	**<0.001**	0.180	(0.116, 0.244)	**<0.001**	0.157	(0.089, 0.224)	**<0.001**
Z-score of birth body weight	0.378	(0.203, 0.554)	**<0.001**									
Small for gestational age	-0.623	(-1.091, -0.156)	**0.009**				-0.150	(-0.632, 0.331)	0.539	-0.163	(-0.642, 0.316)	0.504
Sex	0.035	(-0.236, 0.307)	0.798	-0.172	(-0.44, 0.096)	0.208	-0.165	(-0.435, 0.104)	0.228	-0.145	(-0.414, 0.124)	0.289
Cesarean section	-0.318	(-0.589, -0.048)	**0.021**									
Antenatal steroid	-0.067	(-0.481, 0.348)	0.752									
Maternal education (≥ college)	0.117	(-0.158, 0.391)	0.403									
Preeclampsia	-0.385	(-0.720, -0.050)	**0.024**									
Gestational diabetes	0.602	(-0.073, 1.277)	0.080									
Multi-gestation	0.148	(-0.153, 0.449)	0.334									
Surfactant for RDS	-0.389	(-0.695, -0.084)	**0.013**									
Early onset sepsis	0.720	(-0.157, 1.597)	0.107									
Surgery for PDA	-0.423	(-0.797, -0.050)	**0.027**									
Surgery for ROP	-0.653	(-1.147, -0.158)	**0.010**									
Chronic lung disease	-0.524	(-0.811, -0.237)	**<0.001**									
Postnatal steroid	-0.899	(-1.370, -0.429)	**<0.001**							-0.509	(-1.002, -0.016)	**0.043**
Late onset sepsis	-0.055	(-0.416, 0.306)	0.765									
Necrotizing enterocolitis	-0.121	(-0.774, 0.532)	0.715									
Total thyroxine, µg/dL	-0.015	(-0.084, 0.055)	0.680	-0.112	(-0.186, -0.038)	**0.003**	-0.110	(-0.184, -0.036)	**0.004**	-0.112	(-0.185, -0.038)	**0.003**

Linear regression was performed for each dependent variable and independent variable. Statistical significance was assumed for $p < 0.05$ (indicated in bold). PDA: hemodynamic significant patent ductus arteriosus. CLD: chronic lung disease. CI: confidence interval; LB: lower border; UB: upper border

Supplemental Table S6. Secondary outcome: dependence of body mass index, body weight, body height at 24 months old on clinical variables: multivariate analyses

	Multivariate Model for BMI			Multivariate Model for BW			Multivariate Model for BH		
	β	95%CI	p	β	95%CI	p	β	95%CI	p
Gestational age	0.160	0.036, 0.283	**0.011**	0.182	0.056, 0.308	**0.005**	0.246	-0.043, 0.535	0.095
Z-score of birth body weight	0.560	0.297, 0.823	**<0.001**	0.746	0.477, 1.014	**<0.001**	1.319	0.703, 1.935	**<0.001**
Sex	0.265	-0.075, 0.606	0.127	0.513	0.164, 0.861	**0.004**	1.190	0.39, 1.99	**0.004**
Preeclampsia	-0.222	-0.701, 0.256	0.361	-0.317	-0.808, 0.173	0.204	-0.505	-1.631, 0.622	0.379
Surfactant for RDS	-0.223	-0.621, 0.175	0.271	-0.195	-0.602, 0.211	0.345	-0.065	-0.998, 0.869	0.891
Surgery for PDA	-0.233	-0.747, 0.281	0.373	-0.025	-0.549, 0.5	0.926	0.716	-0.488, 1.92	0.243
Surgery for ROP	-0.328	-1.018, 0.361	0.349	-0.369	-1.073, 0.335	0.303	-0.698	-2.314, 0.918	0.396
Postnatal steroid	-0.557	-1.227, 0.112	0.102	-0.815	-1.498, -0.132	**0.020**	-1.607	-3.175, -0.038	**0.045**
Total thyroxine, μg/dL	-0.136	-0.231, -0.041	**0.005**	-0.088	-0.185, 0.01	0.078	0.070	-0.154, 0.293	0.540

RDS: respiratory distress syndrome; PDA: hemodynamic significant patent ductus arteriosus; ROP: retinopathy of prematurity; BMI: body mass index; BW: body weight; BH: body height. Linear regression was performed for each dependent variable and independent variable. Statistical significance was assumed for $p < 0.05$ (indicated in bold).

Supplemental Table S7. Secondary outcome: dependence of body weight z-scores and body height z-score at 24 months old on clinical variables: multivariate analyses

	Multivariate Model for zBW			Multivariate Model for zBH		
	β	95%CI	p	β	95%CI	p
Gestational age	0.126	0.032, 0.219	**0.009**	0.070	-0.02, 0.161	0.128
Z-score of birth body weight	0.576	0.376, 0.775	**<0.001**	0.421	0.228, 0.615	**<0.001**
Sex	-0.084	-0.343, 0.174	0.521	-0.078	-0.329, 0.173	0.539
Preeclampsia	-0.217	-0.58, 0.145	0.239	-0.171	-0.523, 0.181	0.339
Surfactant for RDS	-0.156	-0.458, 0.146	0.309	-0.047	-0.34, 0.246	0.753
Surgery for PDA	0.050	-0.34, 0.439	0.802	0.265	-0.114, 0.643	0.170
Surgery for ROP	-0.258	-0.78, 0.265	0.333	-0.139	-0.647, 0.369	0.590
Postnatal steroid	-0.740	-1.248, -0.233	**0.004**	-0.654	-1.147, -0.162	**0.009**
Total thyroxine, μg/dL	-0.048	-0.12, 0.024	0.189	0.030	-0.04, 0.1	0.396

RDS: respiratory distress syndrome; PDA: hemodynamic significant patent ductus arteriosus; ROP: retinopathy of prematurity; z BW: body weight z score; zBH: body height z score. Statistical significance was assumed for $p < 0.05$ (indicated in bold).
